# Osteoporosis: From Molecular Mechanisms to Therapies 3.0

**DOI:** 10.3390/ijms222312725

**Published:** 2021-11-25

**Authors:** Chih-Hsin Tang

**Affiliations:** 1 Department of Pharmacology, School of Medicine, China Medical University, Taichung 40402, Taiwan; chtang@mail.cmu.edu.tw; Tel.: +886-22052121 (ext. 7726); 2 Chinese Medicine Research Center, China Medical University, Taichung 40402, Taiwan; 3 Department of Biotechnology, College of Health Science, Asia University, Taichung 41354, Taiwan

**Keywords:** osteoporosis, treatment, prevention, molecular mechanisms, signaling pathways

## Abstract

Osteoporosis is a common skeletal disorder that occurs as a result of an imbalance between bone resorption and bone formation, with bone breakdown exceeding bone building. Bone resorption inhibitors, e.g., bisphosphonates, have been designed to treat osteoporosis. Teriparatide, an anabolic agent, stimulates bone formation and corrects the characteristic changes in the trabecular microarchitecture. However, these drugs are associated with significant side effects. It is therefore crucial that we continue to research the pathogenesis of osteoporosis and seek novel modes of therapy. This editorial summarizes and discusses the themes of the six articles published in our Special Issue “Osteoporosis: From Molecular Mechanisms to Therapies 3.0”, a continuation of our 2020 Special Issue "Osteoporosis: From Molecular Mechanisms to Therapies". These Special Issues detail important global scientific findings that contribute to our current understanding of osteoporosis.

Osteoporosis is a common skeletal disorder that occurs as a result of an imbalance between bone resorption and bone formation, with bone breakdown exceeding bone building. Bone resorption inhibitors, e.g., bisphosphonates, have been designed to treat osteoporosis. Teriparatide, an anabolic agent, stimulates bone formation and corrects the characteristic changes in the trabecular microarchitecture. However, these drugs are associated with side effects. It is therefore crucial that we continue to research the pathogenesis of osteoporosis and seek novel modes of therapy.

All of the articles submitted to us for this Special Issue, “Osteoporosis: From Molecular Mechanisms to Therapies 3.0”, underwent a rigorous peer review process, and ultimately, six met our inclusion criteria. Four of these articles detail original research into (i) a novel molecular mechanism that represses high-fat diet-mediated osteoporosis and body adiposis, (ii) evidence favoring the targeting of visfatin in the treatment of metastatic chondrosarcoma, (iii) a potential role for the bioactive compound paeonoside in the treatment of osteoporosis, and (iv) the use of a novel mutant RANKL that can effectively treat RANKL-induced osteoclastogenesis in both the cellular and preclinical modeling of osteoporosis, apparently without the toxicity that commonly accompanies other antiosteoporotic drugs. This Special Issue also contains two reviews, the first of which discusses the potential therapeutic benefits of using melatonin in the treatment of osteoporosis and osteolytic bone metastasis, while the second describes in vitro and in vivo evidence for the microRNA regulation of the bone/fat formation switch in bone marrow, which has important implications for the homeostasis of bone marrow. These six articles are discussed below.

(i) A novel molecular mechanism that represses high-fat diet-mediated osteoporosis and body adiposis: Lian and colleagues describe how microRNA-29a controls leptin signaling and the brown/beige adipocyte formation of osteogenic progenitor cells and thereby preserves bone anabolism, counteracting whole-body fat overproduction in miR-29a transgenic mice on a high-fat diet [1].

(ii) A potential role for the bioactive compound paeonoside in the treatment of osteoporosis: Preliminary evidence from Park and colleagues demonstrates that paeonoside increases osteoblast differentiation and mineralization [2]. These researchers suggest that paeonoside may be developed as an anabolic compound for the prevention and treatment of bone diseases [2].

(iii) The use of a novel mutant RANKL that can effectively treat RANKL-induced osteoclastogenesis in both cellular and preclinical modeling of osteoporosis, without the toxicity that commonly accompanies other antiosteoporotic drugs: Jang and colleagues constructed a mutant RANKL protein that effectively inhibited RANKL-induced osteoclastogenesis in mice with osteoporosis via the stimulation of GSK-3β phosphorylation as well as the inhibition of NFATc1 translocation, mRNA TRAP and OSCAR expression, TRAP activities, and bone resorption [3].

The review articles discuss molecular and clinical evidence that support the use of melatonin for stimulating osteoblastogenesis, inhibiting osteoclastogenesis, and inducing apoptosis in osteoclasts as well as for suppressing osteolytic bone metastasis [4] and also provide insights into the mechanisms underlying bone growth defects and disordered bone metabolism in bone marrow, indicating that future microRNA-based therapies may one day regulate bone metabolism by modulating the bone/fat switch in bone marrow [5].

We hope that this Special Issue will inspire researchers to continue their explorations into novel osteoporosis prevention and treatment strategies. 
